# Airway hyper-responsiveness in lipopolysaccharide-challenged common marmosets (*Callithrix jacchus*)

**DOI:** 10.1042/CS20130101

**Published:** 2013-09-19

**Authors:** Christoph Curths, Judy Wichmann, Sarah Dunker, Horst Windt, Heinz-Gerd Hoymann, Hans D. Lauenstein, Jens Hohlfeld, Tamara Becker, Franz-Josef Kaup, Armin Braun, Sascha Knauf

**Affiliations:** *Department of Airway Immunology, Fraunhofer Institute for Toxicology and Experimental Medicine, Nikolai-Fuchs-Strasse 1, 30625 Hannover, Germany; †Biomedical Research in Endstage and Obstructive Lung Disease Hannover (BREATH), Member of the German Center for Lung Research, Germany; ‡Pathology Unit, German Primate Center, Leibniz-Institute, Kellnerweg 4, 37077 Göttingen, Germany; §Department of Aerosol Physics, Fraunhofer Institute for Toxicology and Experimental Medicine, Nikolai-Fuchs-Strasse 1, 30625 Hannover, Germany; ∥Department of Immunology, Hannover Medical School (MHH), Carl-Neuberg-Strasse 1, 30625 Hannover, Germany

**Keywords:** airway hyper-responsiveness, lipopolysaccharide, lung-function measurement, lung resistance, marmoset, non-human primate, AHR, airway hyper-responsiveness, *C*_dyn_, dynamic compliance, COPD, chronic obstructive pulmonary disease, CRP, C-reactive protein, EF_50_, mid-expiratory flow, LPS, lipopolysaccharide, MCh, methacholine, MV, minute volume, NHP, non-human primate, PD, provocative dose, *P*_oes_, oesophageal pressure, *P*_TP_, transpulmonary pressure, *R*_L_, lung resistance, *V*_T_, tidal volume

## Abstract

Animal models with a high predictive value for human trials are needed to develop novel human-specific therapeutics for respiratory diseases. The aim of the present study was to examine lung-function parameters in marmoset monkeys (*Callithrix jacchus*) that can be used to detect pharmacologically or provocation-induced AHR (airway hyper-responsiveness). Therefore a custom-made lung-function device that allows application of defined aerosol doses during measurement was developed. It was hypothesized that LPS (lipopolysaccharide)-challenged marmosets show AHR compared with non-challenged healthy subjects. Invasive plethysmography was performed in 12 anaesthetized orotracheally intubated and spontaneously breathing marmosets. Pulmonary data of *R*_L_ (lung resistance), *C*_dyn_ (dynamic compliance), EF_50_ (mid-expiratory flow), *P*_oes_ (oesophageal pressure), MV (minute volume), respiratory frequency (*f*) and *V*_T_ (tidal volume) were collected. Measurements were conducted under baseline conditions and under MCh (methacholine)-induced bronchoconstriction. The measurement was repeated with the same group of animals after induction of an acute lung inflammation by intratracheal application of LPS. PDs (provocative doses) of MCh to achieve a certain increase in *R*_L_ were significantly lower after LPS administration. AHR was demonstrated in the LPS treated compared with the naïve animals. The recorded lung-function data provide ground for pre-clinical efficacy and safety testing of anti-inflammatory substances in the common marmoset, a new translational NHP (non-human primate) model for LPS-induced lung inflammation.

## INTRODUCTION

Lung-function tests are a substantial part of efficacy and safety testing of pharmaceuticals against inflammatory lung diseases such as asthma and COPD (chronic obstructive pulmonary disease) [1,2]. Invasive and non-invasive approaches are described for rhesus (*Macaca mulatta*) and cynomolgus (*Macaca fascicularis*) monkeys [3–9]. In contrast, lung-function tests in the common marmoset (*Callithrix jacchus*), our model species for neutrophilic human airway diseases [10], are virtually non-existent in the literature. The marmoset as a New World monkey species represents a new translational NHP (non-human primate) model for human inflammatory airway diseases. In comparison with Old World monkeys (e.g. rhesus macaques) marmosets provide a better cost–benefit ratio. NHPs reflect the human situation in terms of anatomy, physiology and immunology, and hence represent a highly homologous model species [11–13]. It is predicted that NHP models provide a sophisticated approach towards pre-clinical testing of newly developed human-specific biopharmaceuticals [14,14a].

Yet, only whole-body plethysmography was previously conducted in marmosets. MV (minute volume) and respiratory frequency were the only parameters that have been measured and published so far [15]. Invasive lung-function measurement, however, acquires respiratory flow and *P*_oes_ (oesophageal pressure) data which leads to additional lung-function readout parameters such as *C*_dyn_ (dynamic compliance) and *R*_L_ (lung resistance).

The aim of the present study was to examine lung-function parameters in marmoset monkeys that can be used to detect pharmacologically or provocation-induced AHR (airway hyper-responsiveness). Therefore, we developed a custom-made lung-function device with simultaneous inhalation that allows application of defined aerosol doses during measurement. It was hypothesized that LPS (lipopolysaccharide)-challenged marmosets show AHR compared with non-challenged healthy subjects.

## MATERIALS AND METHODS

### Animals and housing conditions

A total of six female and six sterilized male adult marmosets were utilized for lung-function measurement and corresponding procedures (Supplementary Table S1 at http://www.clinsci.org/cs/126/cs1260155add.htm). Animals were 3.4±0.5 years of age and their body weight was 394±24 g at the start of the experiment (values are means±S.E.M.).

Care and housing conditions at the Encepharm GmbH/German Primate Centre, Göttingen, Germany, fulfilled German and European regulations: national animal protection act (§7-9/TierSchG/7833-3) and European Parliament and European Council Directive on the protection of animals used for scientific purposes (2010/63/EU). Experiments were approved by the Lower Saxony Federal State Office for Consumer Protection and Food Safety, Germany (reference number AZ 33.14-42502-04-084/09).

Animals were housed pairwise at 299.2±1.5 K room temperature, 60–80% relative humidity and 12 h circadian rhythm. Diet comprised pellets suitable for marmosets (ssniff Spezialdiäten), fruits, vegetables and water *ad libitum*.

### Blood sampling

**Figure 1:**
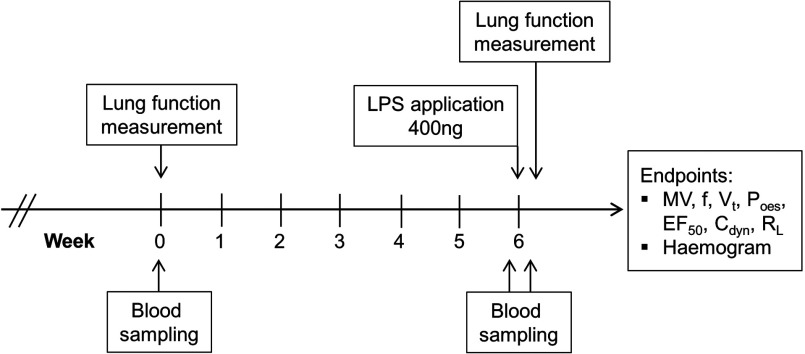
Scheme of the study design Lung-function measurement comprised recording basic data, MCh-induced bronchoconstriction and treatment by salbutamol. Blood samples were taken before the start and pre- and post-LPS application. LPS served to induce lung inflammation prior to second lung-function measurement.

In conscious animals, 1 ml of blood was taken from the animal's femoral vein at days 0, 42 and 43 (Figure 1). The puncture site was disinfected using 70% (v/v) ethanol. Blood was collected from the vena femoralis with a sterile 1 ml syringe and a 26 guage needle. After withdrawal of the needle the vein was gently compressed proximal to the puncture site for approximately 2 min to avoid haematoma. One fraction of the whole blood (600 μl) was transferred to a blood collection tube (EDTA-VACUETTE®, Greiner) and centrifuged at 277 K for 20 min at 1100 ***g*** (Labofuge^GL^, Heraeus). Serum was collected and stored until further processing at 253.2 K for subsequent serological analysis (Dimension® Xpand® Plus, Siemens). The second fraction of whole blood (400 μl) was transferred to an EDTA-coated tube (K2E S-Monovette®, Sarstedt) and used for haematological analysis (Advia® 2120, Siemens). A list of haematological and serological readout parameters can be found in Supplementary Table S2 (at http://www.clinsci.org/cs/126/cs1260155add.htm).

### Lung-function testing for measurement of AHR

The custom-made invasive lung-function measuring station with inhalation system for marmosets has been constructed by Fraunhofer ITEM based on the technique for rodents published previously [2,16]. The plethysmograph enables pulmonary function testing of anaesthetized, orotracheally intubated and spontaneously breathing marmosets. A dose-control system allows administration of defined doses of pharmacological or provocative aerosols during measurement [16]. Healthy marmosets, before LPS challenge, were used to obtain physiological baseline data of lung function by measuring the appropriate parameters: MV, respiratory frequency (*f*), *V*_T_ (tidal volume), *P*_oes_, EF_50_ (midexpiratory flow), *C*_dyn_ and *R*_L_. Differences of lung-function parameters in response to MCh (methacholine) provocation were recorded. At 6 weeks after initial lung-function testing, animals received LPS intratracheally and were measured a second time 18 h later.

**Figure 2:**
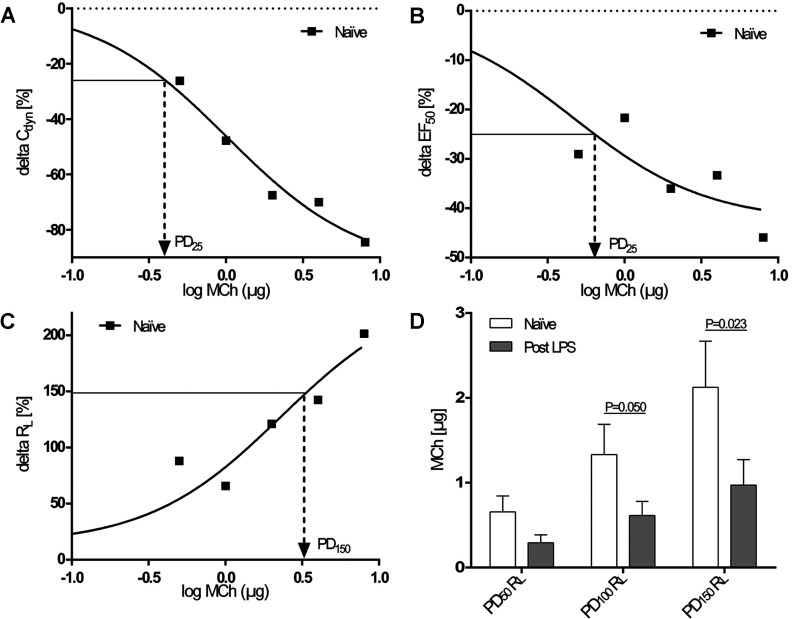
Responsiveness of lung-function parameters to MCh provocation (**A**–**C**) PDs of MCh were calculated for individual dose–response curves, examples of which are shown for Δ*C*_dyn_ (**A**), ΔEF_50_ (**B**) and Δ*R*_L_ (**C**) in naïve marmosets (*R*^2^=0.98, 0.87 and 0.92). Logarithmic MCh values were reversed to obtain PDs. (**D**) PDs of MCh required to increase *R*_L_ 50, 100 and 150% above baseline in naïve animals and 18 h after LPS administration. Results are means±S.E.M., *n*=10 for PD_50_ and PD_100_ and *n*=9 for PD_150_; paired Student's *t* test.

PDs (provocative doses) were defined as the dose of MCh required to alter an individual lung-function parameter to a certain percentage level above or below baseline value. Individual dose–response curves were used to assess PD values. Usually, a three-parameter curve fit was employed to derive PDs (Figures 2A–2C). Animals were removed from provocation test after the MCh dose step at which they reached >150% above baseline *R*_L._ A linear regression was performed to calculate PD values in hyper-reactive animals that reached the 150% *R*_L_ threshold within the first two doses of MCh.

### Procedure

General anaesthesia was initiated by intramuscular injection containing 0.25 mg of diazepam (Diazepam-ratiopharm®, ratiopharm) and 12–18 mg of alphaxalone (Alfaxan®, Vétoquinol) per kg of body weight. Marmosets were orotracheally intubated with a custom-made tube (1.8 mm inner diameter and 9.4 cm length) under visual control utilizing a 75 mm laryngoscope (Classic^+®^, Heine). Intubated animals were immediately connected to the lung-function device via a pneumotachometer linked to a pressure transducer (differential low-pressure transducer DLP 2.5, Hugo Sachs Elektronik, Harvard Apparatus) to determine tidal flow. The spontaneously breathing monkeys were placed on a heating pad to maintain body temperature in a lateral recumbency. A pulse oximeter (MedAir PulseSense VET pulse oximeter, Kruuse) was utilized to monitor HR (heart rate) and blood oxygen saturation. Anaesthesia was maintained with isoflurane (Isofluran Baxter, Baxter Deutschland) applied via orotracheal tube. A probe filled with double-distilled gas-free water was gently inserted into the oesophagus to derive *P*_oes_ at mid-thorax level. This corresponds to *P*_TP_ (transpulmonary pressure). The oesophageal probe was connected to a pressure transducer (P75 Type 379, Hugo Sachs Elektronik, Harvard Apparatus). Amplified analogue pressure transducer signals were digitized using a converter (DT 9804, Data Translation®) at a sampling rate of 250 Hz. Lung-function parameters were recorded and calculated using Notocord-hem™ software (NOTOCORD® Systems) as previously described for rodents [2,17]. Briefly, *V*_T_ was calculated from tidal flow by integration over time. Likewise, MV, EF_50_ (flow at 50% *V*_T_ expired) and respiratory frequency were derived from respiratory flow signals. *R*_L_ was calculated as a quotient of *P*_TP_ and tidal flow, and *C*_dyn_ from the ratio of volume to *P*_TP_ over one breath cycle, using an integration method [17].

**Figure 3:**
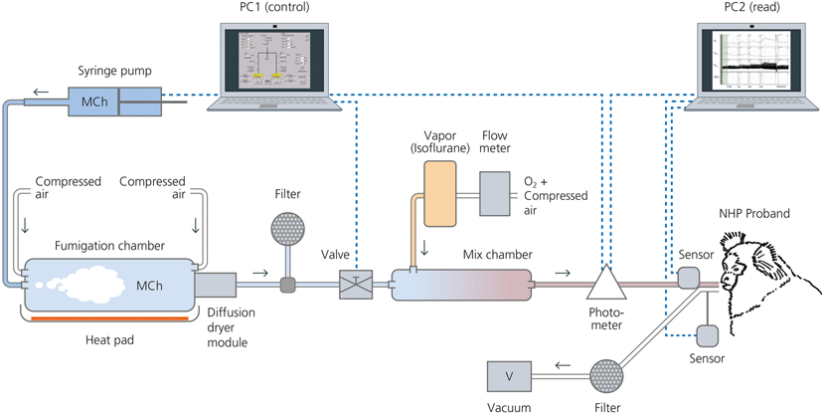
Illustration of the lung-function measuring station with inhalation system

Subsequent to initial baseline measurement bronchial provocation was started. Each animal received defined and stepwise increasing doses of aerosolized MCh, a non-selective muscarinic receptor agonist. MCh solution was prepared with 50 mg/ml acetyl-β-methylcholine chloride (Sigma–Aldrich) diluted in pure water (Ampuwa®, Fresenius Kabi). A perfusor (Vit-Fit polyvalent syringe infusion pump, LAMBDA Laboratory Instruments) controlled syringe injected the MCh solution via a dispersion nozzle (Fraunhofer ITEM) operated with pressurized air into a custom-made evaporation chamber (140 mm diameter and 350 mm length) that was warmed up to 313.2 K. Two mass-flow-controllers (Type 8711, Christian Bürkert) were used for nebulization in a PVC counterflow tube. After this pre-drying of the droplets, aerosol was recooled to 298.2 K and separated from the solvent. A gravimetrically calibrated photometer (Aerosol photometer SMZ-SE, Comde-Derenda) was used to measure MCh concentrations. Custom-made software designed by Fraunhofer ITEM allowed controlled delivery of predefined substance doses by processing the measured signals of aerosol concentration and respiratory MV. The marmoset inhaled the MCh-isoflurane aerosol spontaneously breathing through the pneumotachometer and orotracheal tube. An illustration of the lung-function measuring station with inhalation system is shown in Figure 3.

Baseline lung-function parameters were recorded before MCh provocation. Thereafter, provocation was started with an inhaled MCh mass of 0.5 μg followed by increasing doses of 1, 2, 4 and 8 μg with intermediate breaks of 3 min. The MCh application was stopped when *R*_L_ increased 150% above individual baseline. In a final step, to avoid complications during recovery, animals were treated with a puff (0.1 mg) of the bronchodilator salbutamol (Salbutamol-ratiopharm® N Dosieraerosol, ratiopharm) via orotracheal tube. Recording of lung-function parameters was continued for a minimum of 2 min. Finally, marmosets were removed from the lung-function station, extubated and allowed to recover from anaesthesia, under close monitoring.

### LPS administration

Marmosets were treated with LPS (derived from *Escherichia coli*, serotype 0111:B4) 18 h prior to the second lung-function measurement as previously described for a LPS model [10]. Briefly, induction of anaesthesia was achieved as described for the first lung-function measurement. After orotracheal intubation, 400 ng of LPS dissolved in 200 μl of isotonic saline solution (NaCl 0.9 g/l, WDT) was applied through an intratracheal aerosolizer (MicroSprayer® IA-1C, Penn Century; final dose 1 ng/g of body weight). After removing the aerosolizer, animals were extubated and allowed to recover from anaesthesia.

### Statistics

Statistical analyses were performed using Prism 6.0 (GraphPad Software). Data are shown as means±S.E.M. or medians as indicated. Non-linear three-parameter regression or linear regression models were used to generate individual dose–response curves. These were also necessary to calculate PDs. Differences between data collected before and after LPS application were demonstrated via Wilcoxon test (or paired Student's *t* test for normal distributed samples). *P*≤0.05 was considered statistically significant. Outliers were detected by means of Grubb's test and deleted from further analysis. Dependence of measured parameters from sex, age and weight was analysed via Mann–Whitney *U* Test and Spearman correlation test respectively. An analysis of covariance was performed to test the influence of these covariates on significant changes.

## RESULTS

### Physiological lung-function parameters

Healthy marmosets showed an average MV value of 24.9±2.39 ml/min, a frequency of 26.9±1.87 min^−1^, a *V*_T_ of 0.98±0.11 ml, a *P*_oes_ of 3.90±0.36 cmH_2_O, an EF_50_ of 3.64±0.26 ml/s, *C*_dyn_ of 0.40±0.05 ml/cmH_2_O and *R*_L_ of 0.28±0.03 cmH_2_O·s·ml^−1^ (means±S.E.M., *n*=10).

### Airway responsiveness to MCh provocation

The airway-narrowing effect of applied MCh doses was displayed in changes of relevant bronchoconstriction parameters such as *R*_L_, *C*_dyn_ and EF_50_. At an inhaled dose of 1 μg of MCh *R*_L_ was significantly elevated (0.63±0.11 cmH_2_O·s·ml^−1^), whereas *C*_dyn_ and EF_50_ were significantly decreased (0.26±0.04 ml/cmH_2_O and 1.97±0.42 ml/s) in naïve animals compared with the baseline measurement without MCh (means±S.E.M., *n*=10, *P*=0.003/0.0004/0.0011 for Δ*R*_L_, *C*_dyn_ and EF_50_ respectively, paired Student's *t* test).

Salbutamol administration, which was performed after the highest individual dose step of MCh provocation, resulted in normalization of most lung-function parameters (*R*_L_, *C*_dyn_, EF_50_, MV and *V*_T_). Supplementary Table S3 (at http://www.clinsci.org/cs/126/cs1260155add.htm) shows the same effect on *R*_L_ and EF_50_ compared with 1 μg of MCh, a PD for which all animals were below 150% *R*_L_ increase.

**Figure 4:**
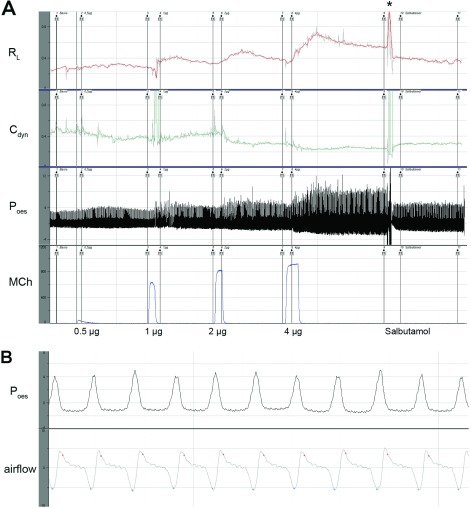
Acquisition of lung-function raw data (**A**) An overview of one complete measurement. From below: photometer signals for applied MCh doses (0.5, 1, 2 and 4 μg). For increasing MCh doses *P*_oes_ (cmH_2_O) increased, whereas *C*_dyn_ (ml/cmH_2_O) decreased and *R*_L_ (cm H_2_O·s·ml^−1^) increased simultaneously. At the end of record, a puff of salbutamol caused a decreasing *P*_oes_, increasing *C*_dyn_ and falling *R*_L_. (**B**) Segment (17 s) of one measurement with individual breaths visible after application of 1 μg of MCh. *P*_oes_ (cmH_2_O) on top and airflow (ml/s) below (snapshots from Notocord-hem™ software; *artefact, manipulation of the orotracheally intubated animal for salbutamol administration).

An individual lung-function recording showing signal traces of the most relevant parameters is shown in Figure 4.

### *R*_L_ as indicator for AHR

PDs of MCh were calculated from individual dose–response curves (examples given in Figures 2A–2C). Compared with naïve non-LPS challenged animals, PD_100_ *R*_L_ and PD_150_ *R*_L_ values were significantly decreased after LPS treatment (Figure 2D), indicating a manifest AHR in animals with acute inflammation (PD_100_, *n*=10 and *P*=0.050; PD_150_, *n*=9 and *P*=0.023, paired Student's *t* test). PD data for EF_50_ and *C*_dyn_ showed no significant differences between naïve and LPS-treated marmosets (results not shown).

### LPS induced a systemic inflammation

The pro-inflammatory systemic effect of intrapulmonary LPS challenge was assessed through haematology and blood chemistry. Blood samples taken before LPS administration contained relative neutrophil and monocyte counts of 40.2±4.5 and 3.2±0.33% (means±S.E.M.). At 18 h after treatment, samples contained 48.1±4.8% neutrophils and 6.8±0.94% monocytes (means±S.E.M.). Thus there was a significant increase in neutrophil and monocyte levels (*n*=10 and *P*=0.005 for neutrophils and *P*=0.003 for monocytes, paired Student's *t* test). Likewise, CRP (C-reactive protein), an acute-phase protein, was significantly increased after LPS treatment (8.5 compared with 9.1 mg/l, *n*=11, median and *P*=0.019, Wilcoxon test).

### Body weight, age and sex

The animals’ body weights were approximately 394±24 g (*n*=12, means±S.E.M.) and were not significantly influenced by treatments. Over the course of the experiment body weight did not change more than 2%. There were single parameters (e.g. *P*_oes_ in naïve animals at baseline) that were influenced by sex, age or weight of the marmosets. However, in no case a significant change of parameters, for example, post- against pre-LPS application or post- against pre-MCh, was attributed to sex, age or weight.

## DISCUSSION

The present paper is the first to report comprehensive lung-function measurement for common marmosets. A previously reported measurement was conducted in a single animal with only MV and frequency recorded [15].

The uptake of inhaled challenge agents or drugs is mainly influenced by breathing frequency and the minute ventilation. Therefore it is important to monitor and control respiratory variables during challenge. Previously, lung-function parameters have been correlated to the concentration of provocative substances [7–9] that as a consequence allows only vague interpretations of deposited dosages. The newly developed lung-function device, however, contains an aerosol generator combined with a computerized dose–control system. Similar to our established models in mice and rats [2,16–18], inhalation of defined dosages of substances such as provocative agents and drugs coincident to lung-function measurement is now possible in the common marmoset, too. This allows a well-defined inhalative application of a pre-selected dose of a single substance.

LPS is part of the outer membrane of Gram-negative bacteria and is known to be a strong inducer of inflammation across species. Applied to the lung, it induces acute lung inflammation and mimics features of airway inflammation of COPD in humans [19,20] and NHPs [10,11,21]. A precise comparison of human and marmoset lung-function data is aggravated because human data are not acquired via invasive plethysmography, but by a forced expiration manoeuvre. In humans, FEV_1_ (forced expiratory volume in 1 s) is reduced after LPS treatment [22,23]. So far, MCh provocation after LPS challenge has only been reported for individuals classified as airway hyper-responsive and asthmatic respectively [24,25]. Baseline data of healthy LPS-challenged and subsequently MCh-provoked humans are therefore virtually non-existent.

Marmosets with acute lung inflammation showed AHR with significantly reduced PD values for MCh provocation. A significant decrease in PD_100_ *R*_L_ and PD_150_ *R*_L_ for LPS-treated animals was observed compared with naïve marmosets (Figure 2D). Animal number is lower for PD_150_ (*n*=9) since one dose–response curve for one individual did not reach a 150% increase in *R*_L_. In contrast with *R*_L_ there were no significant differences for *C*_dyn_ and EF_50_ between the naïve and LPS-treated groups.

Baseline lung-function data of examined marmosets and naïve rats, an animal of comparable size and a classic rodent model species, are within the same range for *R*_L_, *C*_dyn_ and *V*_T_, but EF_50_ is higher in rats [26–29]. MV and frequency are reduced in marmosets compared with rats which could be explained by different anaesthetic protocols. Diazepam [30], alphaxalone [31] and isoflurane [32] are reported to influence breathing patterns in primates. Cholinergic challenge of both naïve rats and investigated marmosets results in a pronounced increase in *R*_L_ and frequency, decrease in *C*_dyn_, and moderate fall in *V*_T_ [26,28,29].

Lung-function measurements were conducted with 12 marmosets. However, the presented data consider ten animals since only complete datasets of lung-function measurement, haematology and blood chemistry were used for statistical analysis (Supplementary Table S1). For animal welfare reasons MCh challenge was stopped at a 150% *R*_L_ increase above baseline level. Like humans, marmosets showed remarkable differences in individual dose responses to cholinergic provocation [23]. Therefore animal numbers decreased with increasing MCh dose. In contrast with most rodent models, marmoset colonies, comparable with the human population, are outbred, which is expressed in such heterogeneous data sets. The test design still has a further advantage since it allows the calculation of PD values at several response levels from all data points of the individual dose–response curve. These are less dependent on variability than a single-dose value. Examples of dose–response curves for percentage change in *R*_L_, *C*_dyn_ and EF_50_ are given in Figures 2(A)–2(C).

As reported previously, there are significant species differences in the potency of bronchoconstriction induced by different mediators [33]. MCh induces a pronounced bronchoconstriction in humans [34] and marmosets [33]. Also, in humans, MCh is favoured over acetylcholine to provoke bronchoconstriction [23,35–37]. In this context, MCh was chosen to assess AHR in marmosets *in vivo* with results supporting our previous findings that humans and marmosets react similarly to MCh provocation [33]. To counteract cholinergic bronchoconstriction at the end of each measurement animals were treated with the bronchodilator salbutamol. This is also practiced for human MCh challenges [35]. Dosing of salbutamol was performed with one puff of salbutamol using a commercial medical inhaler. The applied dosage corresponds to approximately 0.1 mg of salbutamol which resulted in a semi-quantitative analysis of the bronchodilatory effect.

LPS induced a state of acute inflammation as revealed by haematology and the CRP 18 h after LPS challenge. This is consistent with the results of our first study where LPS-challenged marmosets showed similar findings and in addition had significantly higher neutrophil numbers in BALF (bronchoalveolar lavage fluid) [10]. The study design in terms of LPS application in the current experiments was equal to our previous work [10].

A similar systemic inflammatory response with increased blood neutrophil and CRP levels after LPS challenge is observed in humans [19,23,38,39]. Animals in the present study were intentionally not lavaged because remaining fluid would have an effect on the lung-function readout.

The advantage of invasive lung-function measurement compared with non-invasive methods is the assessment of airway resistance and *C*_dyn_, which are sensitive and specific readout parameters for evaluating bronchoconstriction. Orotracheal intubation can be done multiple times, whereas tracheotomization of animals is invasive and does not allow repeated measurements. Pharmacological efficacy and safety studies, however, benefit from multiple lung-function testing [2]. Furthermore, animal numbers are reduced through refining experiments by orotracheal intubation and therefore potential reuse of animals.

Marmosets are strongly diurnal and stress-sensitive [40]. In case animals are overstrained through discomfort or extensive handling, food intake would be affected immediately. The relative high metabolism rate in marmosets would cause a significant and contemporary decrease in body weight. In the present study, marmosets experienced a body weight change not >2%, a strong indicator that the experiment had only marginal, if any, influence on animals’ well-being.

In conclusion, LPS-induced airway inflammation in common marmosets is associated with AHR. A comprehensive set of new readout parameters is presented to further characterize our LPS-induced model for acute lung inflammation in the common marmoset. Reported data from lung-function testing provide a unique opportunity for pre-clinical efficacy testing of anti-inflammatory substances in a relatively inexpensive translational NHP model.

## CLINICAL PERSPECTIVES

• The established invasive lung-function testing in orotracheally intubated marmosets provides ground for pre-clinical safety and efficacy testing of pharmaceuticals in this species. Readout parameters that were previously only accessible in the classic rodent model are now established for a new NHP model.
• There is growing demand of marmosets as the non-rodent ‘second’ species in pre-clinical tests. The technique described to measure lung function in an NHP model will help to support the current need for models with a high predictive power for human clinical trials.
• It furthermore incorporates the 3-Rs. Marmosets are handled similar to human probands and can potentially be used for multiple studies, which reduce the animal numbers.

## Online data

Supplementary data
