# Supplementary material for: Airway hyper-responsiveness in lipopolysaccharide-challenged common marmosets (Callithrix jacchus)
Source: Clin Sci (Lond). 2013 Sep 19;126(Pt 2):155–62. doi: 10.1042/CS20130101 (PMC3793853; doi:10.1042/CS20130101)
Supplement: Supplementary data [file cs1260155add.pdf]

Clinical Science (2014) **126**, 155–162 (Printed in Great Britain) doi: 10.1042/CS20130101

Original Paper

SUPPLEMENTARY ONLINE DATA

Airway hyper-responsiveness in lipopolysaccharide-challenged common marmosets (*Callithrix jacchus*)

Christoph CURTHS*†‡[1], Judy WICHMANN*†‡[1], Sarah DUNKER*†, Horst WINDT†§, Heinz-Gerd HOYMANN*†, Hans D. LAUENSTEIN*†, Jens HOHLFELD*†, Tamara BECKER‡, Franz-Josef KAUP‡, Armin BRAUN*†‖ and Sascha KNAUF*†‡

*Department of Airway Immunology, Fraunhofer Institute for Toxicology and Experimental Medicine, Nikolai-Fuchs-Strasse 1, 30625 Hannover, Germany
†Biomedical Research in Endstage and Obstructive Lung Disease Hannover (BREATH), Member of the German Center for Lung Research, Germany
‡Pathology Unit, German Primate Center, Leibniz-Institute, Kellnerweg 4, 37077 Göttingen, Germany
§Department of Aerosol Physics, Fraunhofer Institute for Toxicology and Experimental Medicine, Nikolai-Fuchs-Strasse 1, 30625 Hannover, Germany
‖Department of Immunology, Hannover Medical School (MHH), Carl-Neuberg-Strasse 1, 30625 Hannover, Germany

Table S1 Overview of the marmoset study population
Experiments utilized 12 animals (body weight at start of experiment; f, female; m, male; x, complete dataset available). For serology abbreviations, refer to Table S2.

				Complete data sets				
						Serology		
Animal number	Age (years)	Body weight (g)	Sex (f/m)	Lung function	Haematology	CRP	CREA, ALT	TP, BUN, TGL
1	8.1	492	m	–	x	x	x	x
2	5.1	390	f	x	x	x	x	x
3	4.2	598	m	x	x	x	–	–
4	4.4	358	m	x	x	x	–	–
5	2.3	373	m	x	x	x	x	–
6	2.7	363	f	x	–	–	x	x
7	2.4	362	f	x	x	x	x	x
8	2.3	458	m	x	–	x	x	x
9	2.3	313	m	x	x	x	x	x
10	2.0	300	f	–	x	x	x	x
11	2.7	330	f	x	x	x	x	x
12	2.8	389	f	x	x	x	x	x

[1]**These authors contributed equally to this work.**

Correspondence: Dr Sascha Knauf (email Sascha.Knauf@item.fraunhofer.de).

© 2013 The Author(s)
The author(s) has paid for this article to be freely available under the terms of the Creative Commons Attribution Licence (CC-BY) (http://creativecommons.org/licenses/by/3.0/) which permits unrestricted use, distribution and reproduction in any medium, provided the original work is properly cited.

Clinical Science www.clinsci.org

Table S2 Parameters determined by automatic serological (Dimension® Xpand® Plus) and haematological (Advia® 2120) measurements of marmoset blood

Data are shown as medians with range; $n = 10$ for haematology.

(a) Serology

Parameter	*n*	Abbreviation	Unit	Before LPS challenge	After LPS challenge
Total protein	9	TP	g/dl	8.1 (6.8–9.0)	7.6 (7.2–9.0)
Creatinin	10	CREA	mg/dl	0.33 (0.20–0.60)	0.44 (0.18–0.68)
Bilirubin	9	BUN	mg/dl	20.0 (13.0–29.0)	24.0 (10.0–36.0)
Tracylglycerols	9	TGL	mg/dl	134.0 (105.0–549.0)	124.0 (69.0–338.0)
Alanine aminotransferase	10	ALT	units/l	15.5 (11.0–50.0)	21.5 (10.0–40.0)
C-reactive protein	11	CRP	mg/l	8.47 (2.90–9.88)	9.11 (3.33–9.72)

(b) Haematology

Parameter	Abbreviation	Unit	Before LPS challenge	After LPS challenge
White blood count	WBC	$10^3/\mu l$	7.1 (4.7–13.4)	7.6 (3.9–12.1)
Red blood count	RBC	$10^6/\mu l$	7.4 (4.7–8.1)	6.9 (3.7–8.0)
Haemoglobin	Hb	g/dl	15.7 (1.5–17.8)	14.7 (7.0–17.6)
Haematocrit	Hct	%	51.7 (31.5–56.6)	49.1 (25.0–56.7)
Mean corpuscular volume	MCV	fl	69.5 (65.5–76.1)	69.7 (65.1–78.2)
Mean corpuscular haemoglobin	MCH	pg	21.5 (1.9–22.4)	21.8 (18.6–23.1)
Mean corpuscular haemoglobin concentration	MCHC	g/dl	31.0 (2.7–31.8)	30.4 (27.4–31.8)
Platelets	PLT	$10^3/\mu l$	469.5 (103.0–692.0)	548.0 (340–712.0)
Neutrophils	Neut	%	40.1 (23.5–67.4)	46.9 (23.2–67.3)
Lymphocytes	Lymph	%	50.9 (24.2–71.1)	40.2 (25.6–64.4)
Monocytes	Mono	%	3.2 (1.8–5.2)	6.6 (1.9–11.1)
Eosinophils*	Eos	%	0.4 (0.0–1.0)	0.8 (0.1–1.1)

* Eosinophil numbers in marmoset monkeys may change when measured in different haematology systems.

Table S3 Marmoset lung function parameters before administration of MCh (baseline), with indicated dose of MCh and after instillation of bronchodilator salbutamol

R_L, C_{dyn}, EF_{50}, P_{oes}, MV, V_T and respiratory frequency (f) are shown as means ± S.E.M. for measurements before LPS challenge ($n = 10$). Salbutamol application was conducted after animals received their highest individual MCh dose. For 1 μg of MCh most of the animals could be included.

Parameter	Baseline	MCh (1μg)	Salbutamol
R_L ($cmH_2O \cdot s \cdot ml^{-1}$)	0.28 ± 0.03	0.63 ± 0.11	0.32 ± 0.06
C_{dyn} (ml/cmH_2O)	0.40 ± 0.05	0.26 ± 0.04	0.15 ± 0.01
EF_{50} (ml/s)	3.64 ± 0.26	1.97 ± 0.42	2.88 ± 0.39
P_{oes} (cmH_2O)	3.90 ± 0.36	8.43 ± 1.23	9.84 ± 0.62
MV (ml/min)	24.9 ± 2.38	31.6 ± 4.88	40.6 ± 3.78
V_T (ml)	0.98 ± 0.11	0.87 ± 0.15	0.92 ± 0.13
f (min^{-1})	26.9 ± 1.87	36.6 ± 3.72	38.2 ± 5.02

Received 4 March 2013/22 July 2011; accepted 23 July 2013

Published as Immediate Publication 23 July 2013, doi: 10.1042/CS20130101

© 2013 The Author(s)

The author(s) has paid for this article to be freely available under the terms of the Creative Commons Attribution Licence (CC-BY) (http://creativecommons.org/licenses/by/3.0/) which permits unrestricted use, distribution and reproduction in any medium, provided the original work is properly cited.
